# Fast nondiffusive response of heat and turbulence pulse propagation

**DOI:** 10.1038/s41598-024-63788-0

**Published:** 2024-06-06

**Authors:** Naoki Kenmochi, Katsumi Ida, Tokihiko Tokuzawa, Yoshinori Mizuno, Ryo Yasuhara, Hisamichi Funaba, Hiyori Uehara, Daniel J. Den Hartog, Mikirou Yoshinuma, Yuki Takemura, Hiroe Igami, Ryoma Yanai

**Affiliations:** 1 National Institute for Fusion Science, Toki, Gifu 509-5292 Japan; 2 The Graduate University for Advanced Studies, SOKENDAI, Toki, Gifu 509-5292 Japan; 3 Wisconsin Plasma Physics Laboratory, University of Wisconsin-Madison, Madison, WI 53706 USA

**Keywords:** Plasma physics, Magnetically confined plasmas

## Abstract

The experimental findings from the Large Helical Device have demonstrated a fast, nondiffusive behavior during the propagation of heat pulses, with an observed increase in speed with reduction in their temporal width. Concurrent propagation of the temperature gradient and turbulence, in a timeframe spanning from a few milliseconds to tens of milliseconds, aligned with the avalanche model. These results indicate that the more spatiotemporally localized the heat and turbulence pulses are, the greater the deviation of the plasma from its equilibrium state, coupled with faster propagation velocity. This insight is pivotal for future fusion reactors, which necessitate the maintenance of a steady-state, non-equilibrium condition.

## Introduction

The advancement toward the realization of fusion power plants is gaining momentum, with significant emphasis on the control of core plasma. Crucial to this endeavor is a comprehensive understanding of plasma parameters such as density and temperature, which are vital for predicting and controlling the high-temperature plasma dynamics. This necessitates a thorough examination of the interaction of the radial profiles of density, velocity, and temperature with the heat flux in magnetically confined plasma, thus resulting in active engagement in transport studies to elucidate these interrelations.

Plasma turbulence can be categorized into two types based on the driving forces governing the particle, momentum, and heat transport, as identified in various studies^1–4^. The first is turbulence locally driven by the local gradients, and the second is spreading turbulence driven at different locations. The transport attributed to the locally driven turbulence is called local transport, whereas that owing to the turbulence spread or modified by turbulence coupling at different locations is called non-local transport. Local transport can be explained by the diffusion coefficient based on Fick’s law, where the radial flux is determined by the local gradient. Conversely, non-local transport is a phenomenon wherein the radial flux is determined globally. Notable instances of observed non-local transport include hysteresis in the gradient-flux relationship^5–7^, see-saw transport^8–11^, and the nondiffusive response prevalent in case of non-local central temperature increases observed in experiments, which characterizes plasma’s reaction to perturbations^12–18^.

For fusion power plants, maintaining stability and steady-state operation in systems far from equilibrium is imperative. This includes managing burst phenomena such as sawtooth oscillations, as well as external heat and particle inputs, similar to those observed in nondiffusive responses. Previous studies have highlighted the significance of responding to deviations from equilibrium states in future fusion reactors. By modulating radial heat fluxes via continuous pellet injection experiments in the Large Helical Device (LHD), the time scale wherein the plasma goes back to the original transport curve is evaluated using the transport potential^19^. Further, the investigation into the dynamical coupling between density gradients and particle transport in various tokamaks (JET, ISTTOK) and stellarators (TJ-II) underscores the importance of a self-regulating mechanism in plasma transport and gradient within fusion devices^20^.

In contrast to passive observations of the nondiffusive response in previous studies, this paper presents results from actively inducing the phenomenon. This study utilized modulated electron cyclotron heating (MECH) experiments in the LHD, while leveraging its advanced diagnostic and heating capabilities, to examine the plasma response. The LHD is particularly suitable for this research owing to its capability for detailed observation of heat and turbulence pulses. In contrast to prior studies that assumed a diffusive nature in examining MECH experiments^21^, this study focused on the plasma response to perturbations, diverging from the traditional approach of investigating transport coefficients. It uncovered new phenomena that deviated from conventional equilibrium transport, thoroughly detailing the fast nondiffusive responses unveiled through the MECH experimental methodology.

## Results

### Experimental set-up

**Figure 1:**
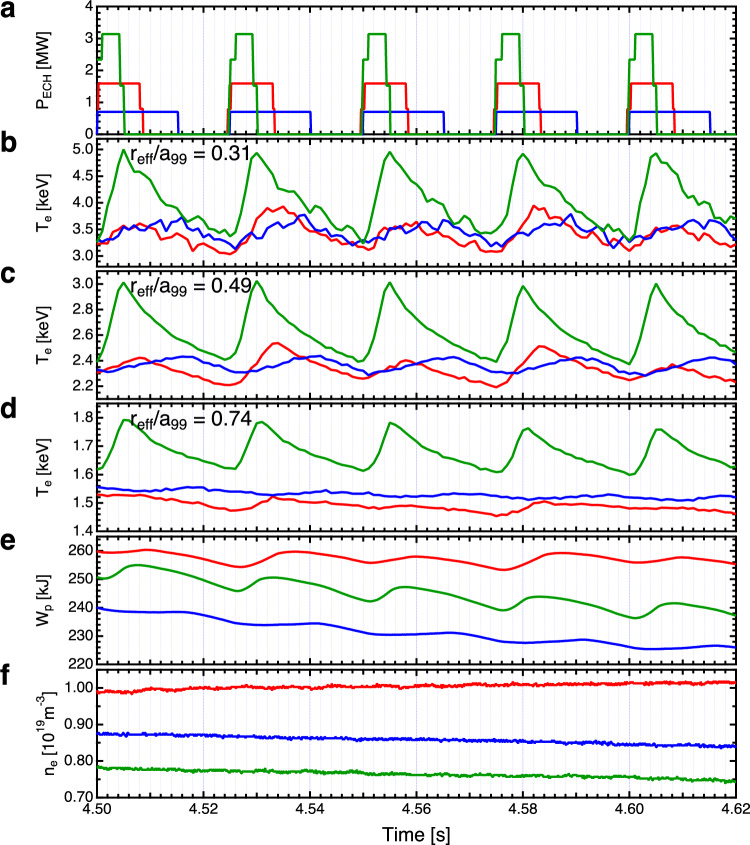
Time evolutions of the (**a**) heating power of ECH, (**b**)–(**d**) electron temperature ($T_e$), (**e**) plasma stored energy, and (**f**) line averaged electron density. The measured positions of the electron temperatures in (**b**), (**c**), and (**d**) are $r_{\text{eff}}/a_{{99}}$ = 0.31, 0.49, and 0.74, respectively. Green, red, and blue correspond to ECH incidence time widths of 4, 8, and 16 ms, respectively.

The experiments were conducted using the LHD, a heliotron-type apparatus for the magnetic confinement of high-temperature plasmas^22^. Figure 1 illustrates the temporal evolution of various parameters, including the injection power of electron cyclotron heating (ECH), electron temperature, plasma stored energy, and line-averaged electron density. The measurement of electron temperature ($T_e$) was performed using an electron cyclotron emission (ECE) radiometer^23^. In these experiments, the focus was to systematically examine the effects of the time scale of turbulence and heat pulse on their propagation velocity. This was achieved in the LHD by manipulating the duration of the heat pulse while maintaining a consistent energy input. This manipulation involved altering the duration and power of the ECH (further details in “Methods” section). The heat was observed to propagate from near the magnetic axis of $r_{\text{eff}}/a_{{99}}$ = 0, wherein the electron cyclotron wave was absorbed, toward the peripheral region. Variations in the ECH pulse width, ranging as 4–20 ms, were implemented, with modulation frequencies set at 25 and 40 Hz. Specifically, pulse widths of 4, 8, 12, and 16 ms, and 5 and 20 ms were used at modulation frequencies of 40 and 25 Hz, respectively. In addition, the injection power was adjusted in tandem with the pulse width to maintain a constant total energy input. This approach ensured the base plasma state remained consistent, thus enhancing the observability of temperature variations during the short pulses. As depicted in Fig. 1b–d, the time evolution of temperature at $r_{\text{eff}} /a_{{99}}$ = 0.31, 0.49, and 0.74 is shown. The temperature changes corresponding to ECH injection time widths of 4, 8, and 16 ms are represented by green, red, and blue lines, respectively. Figure 1e,f demonstrate that both the stored energy and electron density fluctuated within a margin of approximately 10 % even when heating power varied. This indicated that factors other than heating power remain largely unchanged. As shown in Fig. 1b–d, the heat pulses are observed to propagate from the inner to the outer regions of the plasma.

### Non-diffusive propagation of heat and turbulence pulses

**Figure 2:**
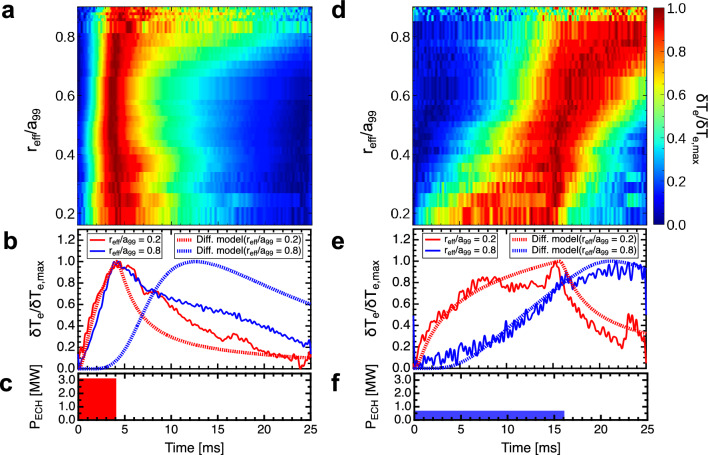
(**a**, **d**) Time evolution of the $T_e$ profiles measured by ECE with the variation normalized between 0 and 1. The pulse widths of ECH in a-c and d-f are 4 ms and 16 ms, respectively. (**b**, **e**) Measured and diffusion-model simulated temperatures for 4 and 16 ms pulse widths, respectively. (**c**, **f**) Time evolutions of ECH injection powers.

**Figure 3:**
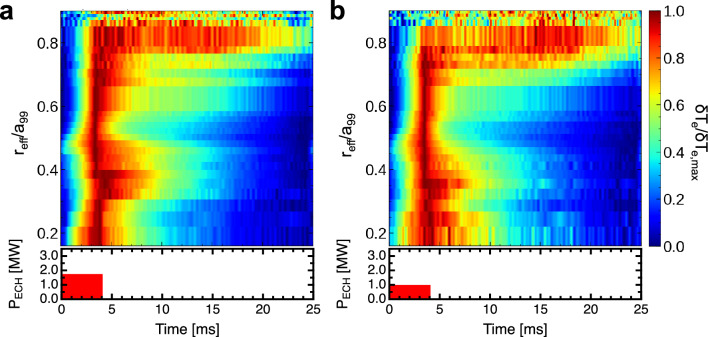
(**a**), (**b**) Time evolution of the $T_e$ profiles measured by ECE with the variation normalized between 0 and 1. Time evolutions of ECH injection powers are shown at the bottom of the figures. The heating powers of ECH in (**a**) and (**b**) are 1.75 and 1 MW, respectively.

Figure 2a,d show the temporal evolution of the $T_e$ profile, as measured by ECE. These profiles were normalized within 0–1 for each location using the conditional averaging technique, corresponding to ECH pulse widths of (a–c)4 and (d–f)16 ms. In this analysis, we define $\delta T_e=T_e-T_{e,\mathrm {min}}$ and $\delta T_{e, \mathrm {max}}=T_{e, \mathrm {max}}-T_{e, \mathrm {min}}$. The figures show the value of $\delta T_{e}/\delta T_{e, \mathrm {max}}$. Herein, $T_{e, \mathrm {min}}$ is the minimum temperature in time at each location and $T_{e, \mathrm {max}}$ is the maximum temperature. Notably, $T_{e, \mathrm {min}}$ is the temperature before heating is applied, that is, the equilibrium temperature. In this process, the $T_e$ data collected via ECE was averaged across each 40 Hz modulation frequency of the ECH to enhance the precision of the analysis. In the two scenarios depicted in Fig. 2, the injection power of the ECH was adjusted while maintaining consistent heating energy conditions. The heating energy injected in one cycle was designed to be constant, and the temperature of the base plasma was kept constant. The experiment revealed a notable trend: shorter pulse widths in heating resulted in faster propagation velocities for both heat and turbulence pulses. Figure 2b,e show the measured and diffusion-model simulated temperatures for 4 and 16 ms pulse widths, respectively. The parameters of the diffusion model were adjusted such that the time evolution aligned with the measured temperature for the experiment of the ECH pulse width of 16 ms (further details on the diffusion model simulation are presented in the “Methods” section). As shown in Fig. 2b, in the experiment with the heating pulse width of 4 ms, a large discrepancy was observed between the measured value at $r_{\text{eff}} /a_{{99}}$ = 0.8 and the model prediction. This indicated that the measured heat propagated to the surrounding area faster than that predicted by the model. Thus, the short pulse propagation phenomenon shown in this experiment deviated significantly from the diffusive response. Conversely, Fig. 3a,b present the heat propagation behavior when the ECH power was altered to 1.75 and 1MW, respectively, with a constant ECH injection time width of 4 ms. Intriguingly, the propagation velocity of the heat pulses remained unaffected by these variations in the heating power. Note that the dramatic and non-periodic structure of the temperature changes in the periphery is due to the fact that the absolute value of the temperature perturbations there is small compared to the overall temperature variation. These findings suggest that the propagation velocity of the heat pulse is primarily influenced by the duration of the heat pulse, rather than its intensity. This indicates the likely absence of transport non-linearities, as the heat pulse velocity does not vary in response to changes in the heat pulse’s intensity.

**Figure 4:**
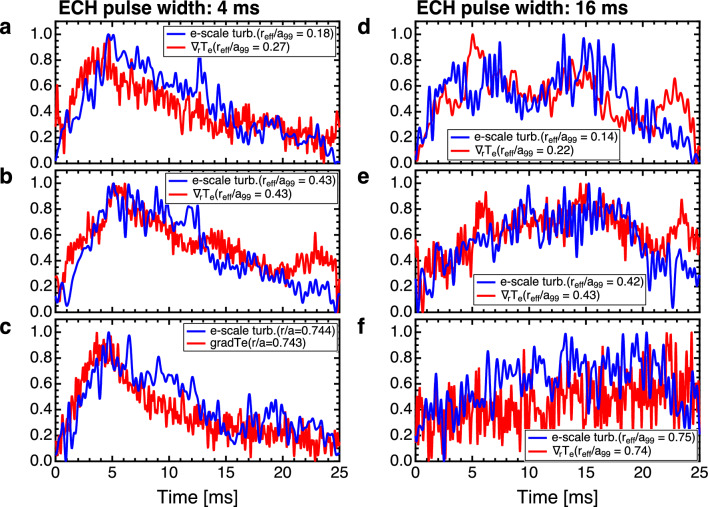
Time evolution of electron scale turbulence (blue) and electron temperature gradient (red) at **a** $r_{\text{eff}}/a_{{99}} \sim 0.2$, **b** $r_{\text{eff}}/a_{{99}} \sim 0.4$, and **c** $r_{\text{eff}}/a_{{99}} \sim 0.7$ for ECH pulse width 4 ms and **d** $r_{\text{eff}}/a_{{99}} \sim 0.2$, **e** $r_{\text{eff}}/a_{{99}} \sim 0.4$, and **f** $r_{\text{eff}}/a_{{99}} \sim 0.7$ for ECH pulse width of 16 ms.

In these experiments, the simultaneous propagation of electron-scale turbulence and heat pulses was observed. Figure 4a–f show the time evolution of electron-scale turbulence as detected through millimeter-wave backscattering measurements for the experiments with ECH pulse widths of 4 and 16 ms. Specifically, the W-band back-scattering diagnostics were used to measure electron-scale turbulence with a high wavenumber of $k_\perp \rho _s \sim 40$ and $k_\perp \sim 40\, \mathrm {cm^{-1}}$, where $k_\perp$ is the fluctuation wavenumber perpendicular to the magnetic field, and $\rho _s$ is the ion gyro radius at the electron temperature^24^. The figures show the sum of fluctuation intensities within the 20–200 kHz range from the back-scattering measurement. The turbulence profiles were assessed by altering the direction of the receiving antenna in successive shots. As the electron temperature gradient is considered as a source of turbulence drive^25^, the radial temperature gradient was calculated based on conditionally averaged electron temperature measurements obtained via ECE. This facilitated a comparative analysis of the temporal evolution of electron-scale turbulence and its response. The relationship between the temperature gradients and electron-scale turbulence was also examined, as illustrated in Fig. 4a–f. The temperature gradients and nearby electron-scale turbulence measurements exhibited similar temporal patterns. To facilitate a comparison, both electron-scale turbulence and temperature gradient values were normalized to a range of 0–1 over time. These findings suggested that temperature gradients and electron-scale turbulence propagated almost simultaneously. In the context of non-local transport, turbulence and heat have been reported to propagate separately under certain conditions, particularly in rapid and abrupt events such as collapse phenomena^3,26^. However, in the observed propagation of heat pulses ranging from a few milliseconds to tens of milliseconds, the turbulent pulses appeared to travel nearly concurrently with the temperature gradient. These results aligned with the conventional understanding embodied in the avalanche model wherein turbulence and temperature gradients propagate simultaneously^27–29^.

## Discussion

**Figure 5:**
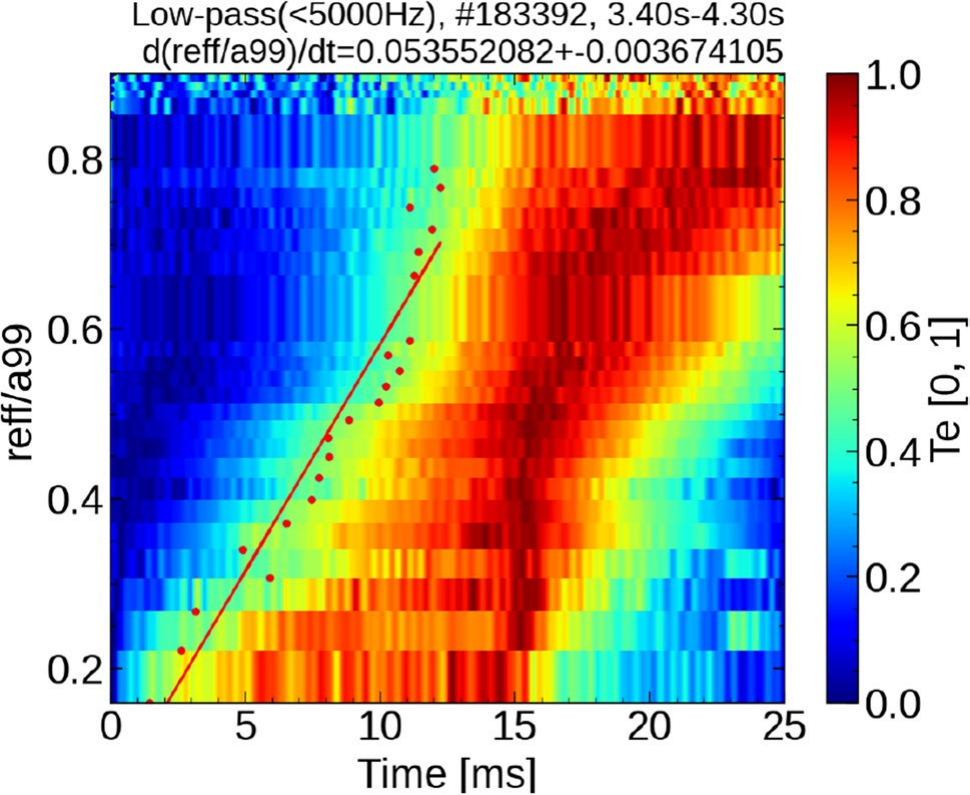
An example of estimation of the propagation velocity of heat pulses. The propagation velocity is evaluated by fitting the time when the normalized temperature reaches a value of 0.5.

**Figure 6:**
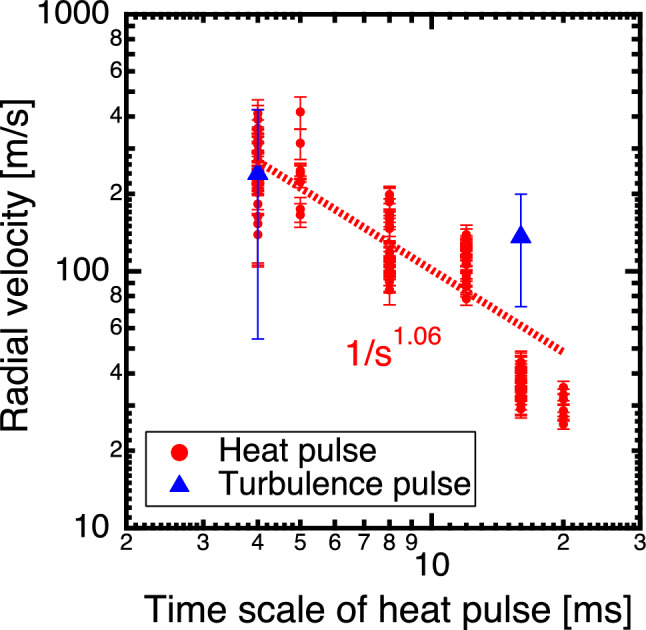
Relationship between the time scale of the heat pulse and the radial propagation velocity of the heat (red) and turbulence (blue) pulses. The red dashed line shows the fitting curve for the propagation velocity of the heat pulse.

As shown in Fig. 5, the radial velocity of the heat pulses is estimated based on their propagation time from $r_{\text{eff}}/a_{{99}}$ = 0.2 to 0.8, measured as the time taken for the pulse to reach half of its peak value. This study did not focus on the behavior observed after turning off the heating injection because the temperature profile shape changes from the equilibrium state and the diffusion characteristics were not considered constant under different heating conditions. Figure 6 presents the relationship between the time scale of the turbulence and heat pulses and their radial propagation velocity, where the time scale was defined by the pulse width of the ECH in the MECH experiments. A notable trend was observed. The smaller the time scale of the turbulence and heat pulses, the greater their velocity, thus exhibiting an approximate power-law relationship. This is evidenced by the propagation velocity of the heat pulse (*v*), which yielded a relationship of $v \propto s^{-1.06}$ with its time scale (*s*), as indicated by the fitted curve in Fig. 6. However, in the experimental results for the heating pulse width of 16 ms shown in Fig. 4f, the increase in the turbulence intensity was greater than that in temperature at approximately *r*_eff_/*a*_99_ = 0.7. This result may be attributed to the velocity of propagation appearing to be faster for the longer pulse width of 16 ms, particularly owing to the mixing of propagation and spatial coupling effects. Consequently, the velocity shown in Fig. 6 was estimated faster than the heat.

The restoration force toward the equilibrium state can be inferred from the speed at which the temperature gradient returns to normal after being disturbed, that is, the propagation speed of heat pulses. In systems far from equilibrium, various phenomena have been reported with changes in the heat propagation speed. For example, in the DIII-D tokamak, short-time scale avalanche events propagate faster than longer-time scale avalanche events^30^. In the electron internal transport barrier (e-ITB) collapse experiment at the LHD, turbulent pulses have been observed to move at a higher velocity than that of heat pulses^26^. The results of this previous study at the LHD aligns with the findings of our study, indicating that heat pulses with a time scale of approximately 1 ms generated by heat avalanches propagated at approximately 1 km/s, consistent with the extension of the power law observed here. The deviation from the equilibrium state is crucial in understanding the propagation of pulses. Short pulses that are spatiotemporally localized, considered far from equilibrium, propagate faster relative to the transport scale. Whereas, longer pulses, closer to an equilibrium state perturbation, exhibit slower propagation velocities, akin to transport speeds. Physically, this implies that longer pulse widths propagate at a transport scale explicable by the local model, whereas shorter pulse widths propagate rapidly owing to spatial coupling with turbulence, indicative of non-local transport. As the profile reverts to its original state, increased turbulence coupling results in exacerbated transport and consequently faster propagation velocity. This study bridges the gap in comprehending the non-local transport mechanism between the radial propagation and the mediator^4^. The observed time scale-dependent propagation velocity challenges existing transport models and provides vital insights into the physics of non-local transport. Understanding the dependence of heat and turbulence propagation velocities on the time width of heat pulses is pivotal for predicting and controlling the dynamic responses in systems far from equilibrium, as maintained by external energy and momentum supplies.

Thus, this investigation revealed that the propagation speed of heat pulses exhibited nondiffusive characteristics, accelerating as their time width decreases, as demonstrated in experiments using the LHD. Concurrently, both the temperature gradient and turbulence propagated simultaneously over a time scale of a few milliseconds to tens of milliseconds, congruent with the avalanche model.

## Methods

### Large helical device

The LHD is a heliotron-type apparatus, that is, a toroidal structure utilizing non-axisymmetric coils. These coils generate an average poloidal field vital for the magnetic confinement of high-temperature plasmas. Key parameters include a major radius at the magnetic axis of $R_\text{ax} = 3.6$ m, an average minor radius of $a=0.6$ m, and a magnetic field at the axis reaching up to 2.75 T^22^. The LHD features a total of five gyrotrons, divided into two and three units operating at 77 and 154 GHz, respectively. Each gyrotron can deliver a maximum heating power of 1 MW, primarily for electron heating^31^. The overall heating power is varied by adjusting the number and combination of these gyrotrons, which are concurrently injected into the plasma.

### Modulated electron cyclotron heating with various duty ratios

The duty ratio *D* of MECH experiment is defined as follows:

1 $$\begin{aligned} D = \frac{\Delta t_{ON}}{\Delta t_{ON}+\Delta t_{OFF}} \end{aligned}$$

where $\Delta t_{ON}$ and $\Delta t_{OFF}$ are the time widths with and without heating in the modulated injection of ECH, respectively. For heat transport analysis, MECH experiments utilize heating injection modulated at a constant frequency of $D=0.5$ in a square-wave pattern. However, this study diverged from such conventional use. Instead of employing modulated heating injection with varying *D* for transport analysis, it focused on examining the detailed propagation of heat pulses by varying the heating time width. This was achieved by adjusting the heating time width and employing conditional averaging. For this specific purpose of this study, the heating power was modulated by changing the *D* via the control of the voltage supplied to the gyrotron’s anode. The required voltage signal was fed into the gyrotron’s anode control panel through its external signal input, thus utilizing the function generator WF1974 from NF Techno Commerce. The voltage signals were configured remotely via software developed in the LabVIEW™ graphical programming environment by National Instruments™. While the frequency could technically be set as high as 500 Hz, it is customarily maintained below 50 Hz. This limitation is attributed to the gyrotron requiring several milliseconds to stabilize its oscillation state. The *D* is adjustable within the range of 0–1, thus offering flexibility in the experimental setup.

### Diffusion model simulation

**Figure 7:**
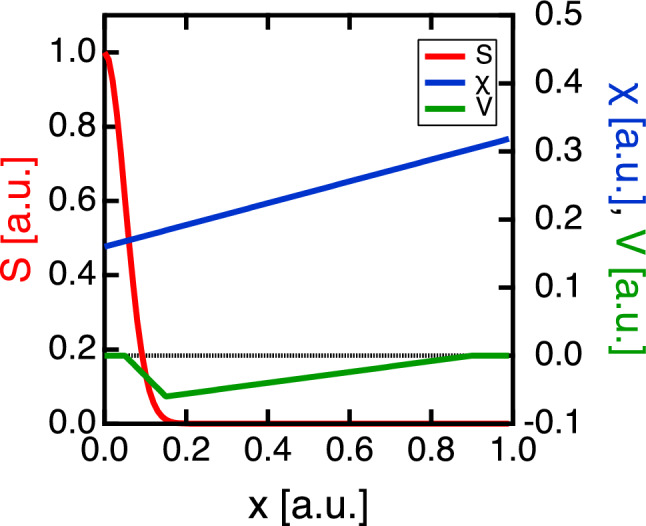
Profiles of *S*, $\chi$, and *V* in the diffusion model simulation used in this study. The profiles are set to reproduce the time variation of the temperature in the experiment with a heating pulse width of 16 ms.

To investigate the mechanism through which the heating time affected the propagation velocity of the thermal pulse, a comparison was made with the results predicted by the diffusion equation. One-dimensional thermal transport equation in cylindrical coordinate for the electron temperature *T* is

2 $$\begin{aligned} n_0\frac{\partial T}{\partial t} + \frac{n_0 T}{\tau } = -\frac{1}{x}\frac{\partial(x q_x)}{\partial x} + S, \end{aligned}$$

where $x,\ n_0,\ \tau ,\ q_x$ and *S* are the radial coordinate, unperturbed electron density, damping term, radial heat flux, and the source term, respectively^32^. The radial heat flux is defined as

3 $$\begin{aligned} q_x = -n_0\chi \nabla T + n_0VT \end{aligned}$$

where $\chi$ and *V* are the diffusion coefficient and convective velocity, respectively. Each of them is regarded to be a function of *T* and $\nabla T$, and can be a function of radius. We solved the transport equation Eq. 2 using the radial heat flux Eq. (3) employing the “Crank–Nicolson method”^33,34^ in the cylindrical coordinate. Here $\tau$ is treated as a spatially uniform finite value. The given radial profiles of $\ S,\ \chi$ and *V* for the simulation are shown in Fig. 7. Each profile was set to reproduce the time evolution of the temperature profile for a heating time width of 16 ms.

### High-k back-scattering measurement

In the LHD, a 90 GHz W-band millimeter-wave back-scattering system is employed to measure electron scale turbulence ($k_\perp \rho _s \sim 40$)^24^. This system is crucial for accurately detecting the intensity of the scattered signal, which correlates with the square of the fluctuations in electron density within the plasma. To achieve high spatial resolution in these measurements, a collinear focusing optical antenna equipped with a metallic lens is installed within the LHD’s vacuum vessel. This setup ensures that the beam diameter is maintained under 40 mm. The scattering volume’s estimated size is approximately 105 and 135 mm at the plasma’s edge and core, respectively. This dimension corresponds to a length of approximately 0.1–0.2 in $r_\text{eff}/a_{99}$. The design of the collinear antenna facilitates the adjustment of the observation position, ranging from the plasma core to its edge, via a remote steering mechanism. In addition, the millimeter-wave heterodyne detection circuit within this system incorporates a modulation function. This function is pivotal in distinguishing and identifying noise components in the scattered signal. These noise components are primarily attributed to the cyclotron radiation emitted by electrons from the plasma, rendering this feature essential for precise signal analysis.

## Data Availability

The raw data were generated at the LHD facility. The data supporting the findings of this study are available in the LHD experiment data repository at https://doi.org/10.57451/lhd.analyzed-data.
